# A pilot study of the impact of an exercise intervention on brain structure, cognition, and psychosocial symptoms in individuals with relapsing-remitting multiple sclerosis

**DOI:** 10.1186/s40814-021-00806-2

**Published:** 2021-03-08

**Authors:** Chantel D. Mayo, Laureen Harrison, Kristen Attwell-Pope, Lynneth Stuart-Hill, Jodie R. Gawryluk

**Affiliations:** 1 Department of Psychology, University of Victoria, PO Box 1700, STN CSC, Victoria, British Columbia Canada; 2 Institute on Aging and Lifelong Health, University of Victoria, Victoria, British Columbia Canada; 3 BC SUPPORT Unit, Victoria, British Columbia Canada; 4 Neurology Department, Island Health, Victoria, British Columbia Canada; 5 School of Exercise Science, Physical Health and Education, University of Victoria, Victoria, British Columbia Canada; 6 Division of Medical Sciences, University of Victoria, Victoria, British Columbia Canada

**Keywords:** Multiple sclerosis, Diffusion tensor imaging, Exercise, Cognition, Fatigue, Depressed mood, Symptom management

## Abstract

**Background:**

Despite pharmacological treatment, many individuals with multiple sclerosis (MS) continue to experience symptoms and medication side effects. Exercise holds promise for MS, but changes in brain structure following exercise have not been thoroughly investigated, and important cognitive and psychosocial variables are rarely primary outcomes. The aim of this pilot study was to investigate whether a 12-week exercise intervention would improve white matter integrity in the brain, or cognition, symptoms of fatigue, and depressed mood for individuals with relapsing-remitting MS (RRMS).

**Method:**

Thirteen participants completed 12 weeks of speeded walking. Baseline and post-intervention testing included 3T diffusion tensor imaging (DTI) to assess white matter and neuropsychological testing to assess cognition, fatigue, and mood. Image pre-processing and analyses were performed in functional magnetic resonance imaging of the Brain Software Library.

**Results:**

Post-intervention, there were no significant changes in white matter compared to baseline. Post-intervention, individuals with RRMS performed significantly better on the Symbol Digit Modalities Test (SDMT), reported fewer perceived memory problems, and endorsed less fatigue. Performance was not significantly different on Trails or Digit Span, and there were no significant changes in reports of mood.

**Conclusion:**

Although 12 weeks of speeded walking did not improve white matter integrity, exercise may hold promise for managing some symptoms of RRMS in the context of this study population.

## Feasibility

*What uncertainties existed regarding the feasibility?*

- Would individuals with RRMS who meet the study’s eligibility criteria be able to complete a 12-week exercise intervention?
- Given the lack of exercise interventions in MS that include brain imaging as an outcome measure, can a novel brain imaging method (such as DTI) be used to examine white matter microstructure pre- and post-exercise?

*What are the key feasibility findings?*

- Eligible individuals with RRMS were able to complete the 12-week exercise intervention, with a mean adherence of 98.29%.
- DTI data was acquired pre- and post-intervention for 13 participants. Microstructural white matter alterations in the brain were not detected post-intervention using DTI, but imaging findings may be limited by a small sample size. Additionally, functional brain changes were not examined in the current study.

*What are the implications of the feasibility findings for the design of the future study?*

- It was feasible for individuals with RRMS who met the study’s eligibility criteria to complete the 12-week exercise intervention.
- Future studies with larger sample sizes that employ a multi-modal imaging approach (e.g., include functional MRI alongside DTI) may further clarify the extent to which DTI is an effective means of tracking the efficacy of exercise interventions for individuals with RRMS. Multi-modal imaging approaches would also provide additional information about functional changes in the brain.

## Background

Multiple sclerosis (MS) is a chronic inflammatory disease characterized by focal white matter degeneration and changes in gray matter volume. Given that the lesions associated with MS can be distributed throughout the central nervous system, individuals with MS often experience a constellation of sensory, motor, and cognitive symptoms, as well as issues with fatigue and mood [1]. Because there is currently no cure for MS, and individuals are typically diagnosed in early adulthood, most individuals require treatment for the majority of their lives [2]. Pharmaceuticals are often prescribed for long-term medical management, but these are costly and have variable effectiveness with possible unwanted side effects [3, 4]. Thus, there is a critical need for the implementation of low-cost and side effect-free behavioral interventions to help with symptom management.

In addition to medical management, exercise holds promise for individuals with MS. Exercise has shown benefits in the prevention of cognitive decline in aging populations [5] and in the management of other types of neurological disorders, such as Parkinson’s disease and Alzheimer’s disease [6, 7]. Importantly, exercise interventions can be accessible (e.g., can be completed without expensive equipment; available to diverse socioeconomic populations) and are non-invasive [8].

To date, several reviews and meta-analyses have looked at the clinical impact of exercise interventions for individuals with MS. These studies have provided early evidence that exercise interventions have modest effects on improving some MS symptoms [9, 10]; however, the primary outcomes of these studies have largely been confined to physical measures (e.g., walking, strength, mobility). There is preliminary evidence that exercise may contribute to small improvements in cognition [11], fatigue [8, 12], and mood [13], but these cognitive and psychosocial variables were often not the primary outcome measures.

Currently, a significant limitation in the literature relates to the lack of investigations on changes in brain structure that may result from exercise interventions and relate to improved symptoms of MS. Indeed, the use of neuroimaging techniques, such as magnetic resonance imaging (MRI), to examine the neural effects of exercise in individuals with MS has been identified as a major gap in the literature [14, 15]. MRI is a non-invasive, safely repeatable technique that allows for the examination of brain structure and is typically used in the assessment and diagnosis of MS [16]. One landmark study performed by Leavitt and colleagues [17] found that the 12-week stationary cycling (3 times per week) contributed to the increased hippocampal volumes, increased resting-state functional connectivity, and improved memory performance.

To date, very few studies have examined diffusion tensor imaging (DTI) metrics pre-post exercise intervention. DTI is a type of MRI scan that measures water diffusion in the brain to provide indices of white matter integrity [18, 19]. DTI’s sensitivity to detect microstructural characteristics of white matter makes it an ideal tool to examine possible neuroprotective effects of exercise on individuals with MS [20].

In preparation for a larger study, the goal of this pilot study was to examine whether the implementation of a 12-week speeded walking exercise intervention would improve white matter integrity (as measured by DTI), cognition, or symptoms of fatigue and depressed mood for individuals with relapsing-remitting MS (RRMS). Secondary objectives were related to the feasibility of the pilot trial (e.g., would eligible individuals with RRMS complete the exercise intervention? Can DTI be used to examine white matter microstructure pre- and post-exercise?)

## Methods

### Recruitment

Participants were recruited from the local health authority’s permission to contact program. The permission to contact program connects eligible researchers with individuals who have declared an interest in participating in research. Interested participants contacted the research team via telephone or email, and they were assessed for eligibility. Based on the largest sample available within both funding and access to MRI constraints, 16 individuals were recruited

### Sample size

Sample size was based on the largest sample available both within funding and access to MRI constraints and is consistent with than previous MRI-based intervention studies involving individuals with MS [17, 27].

### Participants

Participants were eligible if they were diagnosed with RRMS, at least 19 years of age, fluent in English, able to walk without assistance or rest for at least 300 m (consistent with Expanded Disability Status Scale score of 4.5 or less), and able to complete study tasks (e.g., exercise intervention, neuropsychological testing) independently. Exclusion criteria included having any MRI contraindications (e.g., metal implants, pacemakers), claustrophobia, and any comorbid neurological disorders. All participants attended pre- and post-intervention MRI and interview appointments, where neuroimaging, neuropsychological testing, and self-report measures were completed.

### Imaging data acquisition

DTI data were collected by a trained MRI technician with one of the study team members present at West Coast Medical Imaging (Victoria, BC) on a 3T GE Signa Pioneer MRI scanner pre- and post-intervention. The images were acquired with an EPI sequence, axially, with the following parameters: TR = 8000 ms, TE = 101 ms, flip angle = 90, 52 slices, voxel size = 1.4 × 1.4 × 2.0 mm. There were 48 images acquired for each scan: 45 diffusion-weighted images (*b* = 1000 s/mm^2^) and 3 non-diffusion-weighted images (*b* = 0 s/mm^2^). The acquisition took approximately 6 min.

### Neuropsychological testing and self-report measures

Appointments took place at the University of Victoria. Neuropsychological and self-report measures were administered by a trained graduate student in clinical neuropsychology. Pre- and post-intervention neuropsychological testing was conducted using the Symbol Digit Modalities Test (SDMT) [21], Trail Making Test [22], and Digit Span [23] to assess cognition. Participants also completed pre- and post-intervention self-report measures to assess perceived cognitive impairment (attention/concentration, retrospective memory, prospective memory, and planning/organization; Perceived Deficit Questionnaire; PDQ) [24] fatigue (Modified Fatigue Impact Scale; MFIS) [24, 25], and mood (Beck Depression Inventory 2nd edition, BDI-II) [26].

### Speeded walking intervention

The exercise intervention, based on a treadmill walking intervention in a previous pilot trial [27], involved independent speeded walking three times per week at increasing intervals (15 to 35 min) over the course of 12 weeks (36 sessions total). Participants wore activity monitors (Fitbits) to time their sessions, ensure they were reaching a target heart rate zone of at least 50% of maximum predicted heart rate, and encourage adherence. They also logged their walking activity on an exercise tracking form provided to them. Participants were asked not to stop any consistent exercise (e.g., weekly class) in which they were already engaged, nor begin any new exercise that was unrelated to the study. Individuals with MS were not receiving any concurrent physiotherapy during the study period. Participants were asked to report any adverse events to the study team.

### Data analyses

Changes in DTI metrics (fractional anisotropy; FA, mean diffusivity; MD, axial diffusivity; AD, and radial diffusivity; RD), neuropsychological performance, and MS symptoms from pre- to post-intervention were examined. Image pre-processing and analysis was performed using FMRIB Software Library [28]. Eddy Correct was used to correct for eddy current distortions and head movement. Next, brain extraction tool was used to remove the non-brain tissue, and then, brain-extracted images were visually inspected to confirm only non-brain tissue was removed [29]. FA maps were generated using DTIfit and input into Tract-Based Spatial Statistics (TBSS) [30], followed by randomise with threshold-free cluster enhancement. This was repeated for MD, AD, and RD.

Non-imaging group-level demographic and inferential statistical analyses were performed in R Studio. The assumption of normality was assessed using the Shapiro-Wilk test. Deviations from normality were observed for Trails A (*W*=.793, *p*=.006), Trails B (*W*=.866, *p*=.046), Digit Span (*W*=.825, *p*=.014), BDI-II (*W*=.865, *p*=.045), PDQ_Planning&Organization_ (*W*=.822, *p*=.013), but not for SDMT (*W*=.921, *p* =.257), MFIS (*W*=.983, *p*=.990), PDQ (*W*=.930, *p* = .341), PDQ_Attention_ (*W*= .974, *p* = .939), PDQ_ProspectiveMemory_ (*W* =.912, *p* = .194)_,_ PDQ_RetrospectiveMemory_ (*W*=.879, *p* =0.068). Thus, non-parametric Wilcoxon signed-rank tests (Trails A, Trails B, Digit Span, BDI-II, and PDQ_Planning&Organization_) and parametric paired Student *t* tests (SDMT, MFIS, PDQ, PDQ_Attention_, and PDQ_ProspectiveMemory,_ PDQ_RetrospectiveMemory_) and were used to compare post-intervention scores to pre-intervention scores on neuropsychological tests and self-report measures. Corresponding 95% confidence intervals were also calculated for mean paired differences (paired sample *t* tests) and Hodges-Lehmann Estimate (Wilcoxon signed-rank tests). Normed (for age, education, and sex where available) neuropsychological test scores were used.

## Results

### Participants

Sixteen individuals with RRMS were recruited for the study, but three individuals withdrew prior to study completion. Remaining participants included 10 females and 3 males with RRMS (mean age= 58.76 ± 11.07 years). Please see Table 1 for further demographic information. One female’s imaging data was omitted from the DTI analysis due to missing post-intervention scans. Recruitment occurred in June 2018. Data collection took place from August 2018 to November 2018. Participants adherence was 98.29% (mean sessions completed = 35.38 ± 0.77 out of a total of 36 sessions). No adverse events were reported to the study team.

**Table 1:** Participant demographics

**Age (mean ± SD)**	58.76 ± 11.07 years
**Education (mean ± SD)**	15.15 ± 2.08 years
**Gender**	10 females, 3 males
**Time since diagnosis (mean ± SD)**	21.03 ± 16.06 years
**Treated for depression (percent)**	0%
**Co-morbid psychiatric condition (percent)**	0%

### DTI

Post-intervention there were no significant changes in any of the DTI metrics, including FA, MD, AD, or RD (*p*<0.05, corrected for multiple comparisons), compared to baseline. Extracted mean FA, MD, AD, and RD values are reported in Table 2.

**Table 2:** Pre- and post-intervention DTI metrics, including mean fractional anisotropy (FA), and mean, axial, and radial diffusivity (MD; AX; RD; mm^2^/s)

Pre-intervention DTI metrics				Post-intervention DTI metrics			
FA	MD	AX	RD	FA	MD	AX	RD
0.436	0.00081	0.00121	0.00061	0.429	0.00081	0.00119	0.00061
0.499	0.00075	0.00119	0.00053	0.510	0.00073	0.00117	0.00051
0.508	0.00070	0.00112	0.00049	0.493	0.00071	0.00112	0.00051
0.520	0.00070	0.00115	0.00048	0.517	0.00071	0.00116	0.00049
0.483	0.00075	0.00118	0.00054	0.479	0.00075	0.00117	0.00054
0.503	0.00075	0.00122	0.00052	0.505	0.00074	0.00119	0.00051
0.495	0.00075	0.00119	0.00053	0.482	0.00075	0.00119	0.00054
0.498	0.00073	0.00117	0.00051	0.497	0.00074	0.00117	0.00052
0.477	0.00077	0.00120	0.00055	0.463	0.00078	0.00119	0.00057
0.477	0.00076	0.00118	0.00054	0.470	0.00076	0.00117	0.00055
0.516	0.00073	0.00119	0.00050	0.508	0.00073	0.00117	0.00050
0.489	0.00075	0.00119	0.00053	0.477	0.00076	0.00118	0.00054
0.492	0.00075	0.00118	0.00053	0.486	0.00075	0.00117	0.00053

### Cognition, fatigue, and mood

Post-intervention, individuals with RRMS performed significantly better on the SDMT. Additionally, individuals with RRMS perceived fewer prospective memory problems (PDQ) and reported fewer symptoms of fatigue (MFIS) compared to pre-intervention (Table 3).

**Table 3:**
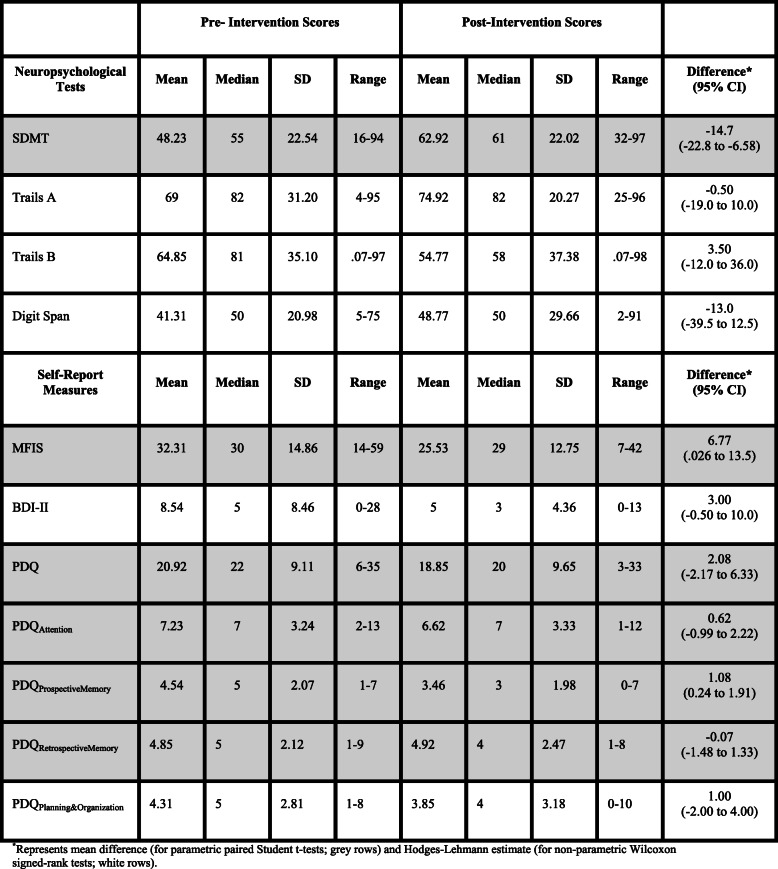
Pre- and post-intervention performance on neuropsychological tests and self-report measures

Post-intervention performance on Trails A, Trails B, and Digit Span was not significantly different than pre-intervention performance. There were also no significant changes in reports perceived problems with attention, retrospective memory, or planning (PDQ) or mood (BDI-II) post-intervention compared to pre-intervention (See Table 3).

## Discussion

This study investigated whether 12 weeks of speeded walking would significantly improve white matter integrity in the brain, cognition, or symptoms of fatigue and depressed mood for individuals with RRMS. It also examined the feasibility of the current pilot trial to be expanded for a future study. Following 12 weeks of speeded walking, individuals with RRMS performed better on a cognitive measure involving processing speed, reported fewer perceived prospective memory problems and significantly less fatigue. There were no changes in white matter integrity, as measured by DTI. Overall, it was feasible for individuals with RRMS to complete the 12-week exercise intervention with excellent adherence. There were also no adverse events reported.

### Brain changes

MS is characterized by demyelination and degeneration of axons, and the use is MRI is integral in clinical assessment and diagnosis [16, 31]. The current pilot study did not observe any white matter changes in individuals with RRMS following 12 weeks of speeded walking, as assessed using DTI. Although there were not significant improvements in white matter integrity, there were also no significant declines in white matter integrity, as may be expected in a demyelinating disorder such as MS [31]. Indeed, several studies have observed longitudinal changes in DTI metrics in individuals with MS. Ontaneda and colleagues [32] observed significant increases in AD in enhancing and chronic lesions over 4 years of follow-up. Additionally, Kolasa and colleagues [33] found increases in FA and AD and decreases in RD in regions of the corpus callosum over 4 years of follow-up.

Changes in brain structure and function following exercise interventions, specifically, have not yet been thoroughly investigated in individuals with MS [15]. Although there have only been a few pilot trials [17, 27] even fewer have used DTI. Recently, Tavazzi and colleagues [34] used TBSS, among other imaging measures, to assess any differences in DTI metrics in 29 individuals following 4 weeks of rehabilitation exercises for MS that involved resistance or endurance training. Similar to our findings, there were no changes in any DTI metrics. Interestingly, they observed increased functional connectivity in the precentral and post-central gyrus at the end of the 4 weeks, but these findings did not hold at a second follow-up 3 months later. Future research in this area would benefit from the use of multi-modal imaging approaches, including functional MRI, DTI, and other structural imaging methods (e.g., T1 and FLAIR scan), to gain a more complete understanding of possible changes in the brain resulting from exercise intervention.

### Cognition

Up to 70% of individuals with MS experience reductions in cognition, especially for tasks that involve information processing, executive function, and memory [35–37]. The current study found that individuals with RRMS performed significantly better on the SDMT following 12 weeks of speeded walking, but not Trails or Digit Span. The SDMT is considered the measure of choice for MS as it has shown to be sensitive to declines in cognition observed in MS and has correlated well with other measures of disease progression, such as atrophy and lesion burden [38, 39]. To date there are mixed findings on the impact of exercise on cognition in MS [11]; while some studies have found exercise improved performance on the SDMT [40] other studies have found no such relationship [17, 41]. As in other psychosocial outcomes, this heterogeneity is possibly attributed to methodological issues, such as diversity in exercise interventions, and cognition not included as a primary outcome [11].

### Fatigue

Fatigue is among the most commonly reported symptoms of MS [42, 43]. The current study found that individuals reported significantly less fatigue after 12 weeks of speeded walking. This result is consistent with findings from other meta-analyses [12]. It is possible that exercise decreases pro-inflammatory cytokines, which are thought to be related to fatigue [6, 44]. Still, there is heterogeneity in findings, perhaps due to the variability in exercise interventions, ranging from endurance to strength training to mixed methods as well as fatigue being included as a secondary outcome measure [8].

### Mood

Low mood is a common symptom of MS, and up to 50% of individuals diagnosed with MS have experienced depression at some point in their lifetime [45, 46]. The current study did not find evidence for significant reductions in reports of depressed mood following 12 weeks of speeded walking. However, it is important to consider that this result may be particular to our study given that at baseline, the majority of participants reported minimal symptoms of depression. It is possible that the impact of exercise on mood may only be evident in individuals with more severe levels of depression. For example, in a meta-analysis, Dalgas and colleagues [13] found that the studies that demonstrated positive impacts of exercise on mood in MS had participants with higher baseline levels of depression.

### Study limitations and future directions

As a pilot trial, the current study is limited by a small sample size, which is comprised exclusively of individuals with RRMS subtype, as has been common in research to date [8]. The current study did not detect any structural white matter changes in the brain following the exercise intervention; this may be due, in part, to a limited sample size, but could also be influenced by additional factors (e.g., that the intensity or duration of the exercise was insufficient). Future research would benefit from larger samples that also include individuals with progressive forms of MS to help clarify this, as well as determine whether imaging, cognitive, and psychosocial outcomes are influenced by MS subtype. Future studies may also benefit from increasing the intensity of the prescribed aerobic exercise, as tolerated by individuals with MS, in accordance with activity recommendations for individuals with MS [47, 48].

The current pilot study also employed a within-persons pre-post design. As research in this area expands beyond pilot trials, the inclusion of additional control groups (i.e., individuals with MS who resume activity as normal) is needed to determine the extent to which exercise may impact the typical progression of pathological changes that occur in MS.

There are also a number of participant characteristics that may be unique to our participant sample. For example, it is also notable that some of our participants were already fairly active. Future research may choose to selective recruit sedentary participants to assess whether exercise has a greater impact for these individuals. Additionally, the majority of our participants were not cognitively impaired at baseline; many performed at or above the 50th percentile. Thus, future research may wish to specifically recruit individuals with cognitive impairment to better assess the impact of exercise on individuals with cognitive impairment.

Furthermore, the extent to which the observed improvements in fatigue and some aspects of cognition may be long lasting is unclear. It is possible that these improvements may eventually plateau, or otherwise decline across time. Thus, it would be useful for future research to track participants longitudinally over a number of years, with frequent follow-up appointments. Additionally, follow-up studies could use a design that captures change over time prior to the intervention, so this change can be compared to change over the course of the intervention. Importantly, given the traditional focus on physical measures (e.g., walking, strength, mobility) future exercise research trials should consider including frequently observed psychosocial and cognitive symptoms as primary outcome measures. Additionally, given the importance of patient-oriented research, future research studies may benefit from the inclusion of minimal clinically important difference scores, which reflect perceived benefits from the patient [49].

Lastly, although the current study did not detect any structural white matter changes in the brain following the exercise intervention, it did not investigate whether any functional changes in the brain took place or whether there were changes in gray matter. There is preliminary evidence from other pilot trials that exercise interventions have contributed to increased resting state functional connectivity in the thalamo-cortical regions [50]. Thus, future research would benefit from a multi-modal imaging approach that also include functional approaches such as functional MRI. It is notable that DTI may have limited clinical use for tracking the efficacy of exercise interventions for RRMS. Thus, multi-modal research may also further clarify the extent to which DTI is an effective means of tracking the efficacy of exercise interventions for people with RRMS.

## Conclusions

Following the implementation of a 12-week walking exercise intervention, individuals with RRMS performed significantly better on a measure of information processing speed, perceived fewer prospective memory problems, and reported fewer problems with fatigue. White matter integrity did not significantly decrease or increase. Together, this suggests that although there is not enough evidence to suggest speeded walking is reparative, 12 weeks of speeded walking holds promise for managing some symptoms for individuals with RRMS. The findings of this study should be interpreted within the context of the study design and limited study population.

## Data Availability

The dataset for the current study may be available from the corresponding author on reasonable request.

## Abbreviations

MS: Multiple sclerosis
RRMS: Relapsing remitting multiple sclerosis
DTI: Diffusion tensor imaging
SDMT: Symbol Digits Modalities Test
PDQ: Perceived Deficits Questionnaire
MFIS: Modified Fatigue Impact Scale
BDI-II: Beck Depression Inventory
FA: Fractional anisotropy
MD: Mean diffusivity
AD: Axial diffusivity
RD: Radial diffusivity
TBSS: Tract-based spatial statistics
